# Consumer perception, mandatory labeling, and traceability of GM soybean oil: evidence from Chinese urban consumers

**DOI:** 10.1080/21645698.2020.1807852

**Published:** 2020-08-23

**Authors:** Mingyang Zhang, Yubing Fan, Chao Chen, Jingxia Cao, Hongshan Pu

**Affiliations:** a School of Business, Development Institute of Jiangbei New Area, Nanjing University of Information Science and Technology, Nanjing, China; b Texas A&M AgriLife Research, Vernon, TX, USA; c College of Economics & Management, Nanjing Agricultural University, Nanjing, China

**Keywords:** Genetically modified foods, mandatory labeling, consumer perception, traceability, China

## Abstract

Consumer preference for the mandatory labeling of genetically modified (GM) foods promotes public support for the implementation of GM food policies. This study analyzes consumers’ preference for the traceability of GM soybean oil. Survey data were collected through a self-administered survey covering 804 randomly sampled urban residents in the eastern, central and western regions of China. Using a logit model, this analysis examines the impacts of influential factors on consumers’ preference for traceability. The results show that about 56.5% of the respondents have a positive preference for the traceability of GM soybean oil. Factors increasing the preference for traceability include a better perception of the attributes of nutrition benefit and potential health risk, perceived inadequacy of simple mandatory labels, more attention paid to food labels, and distrust in the agencies overseeing GM food safety. Enhancing consumers’ perceptions of GM-related attributes and awareness of food labels will help improve the mandatory labeling management of GM foods.

## 1. Introduction

Over the past two decades, the adoption of genetically modified (GM) insect-resistant and herbicide-tolerant technologies has reduced herbicide and insecticide use, which decreases the adverse environmental impacts associated with chemical application. The biotechnology has also facilitated remarkable reduction in tillage operation and fossil fuel use. The greenhouse gas emissions, as a result, greatly decreased in many GM cropping area.^1^ However, with the growing consumption of GM foods, asymmetric information becomes an important issue^2^ and the information asymmetry is primarily due to lack of effective communication about the potential risks.^3^ Worldwide, there are also growing concerns with GM foods, particularly among many civil society groups.^4–7^ Luck et al. reported that about 45% of American consumers have a safety concern with GM foods, of which over 80% are supportive of implementing the mandatory labeling policy on GM products.^8^ The mandatory labeling is considered an effective method to address the issue of asymmetric information.^9^ Currently, the simple GM food label is of limited usefulness to consumers because it only allows to differentiate GM foods from their non-GM counterparts.^10^ Open questions await to be answered with scientific evidence. For example, whether consumers have more stringent requirements for the mandatory labeling of GM foods, such as traceability, and what factors affect consumers’ preference for traceability.

**Figure 1.**
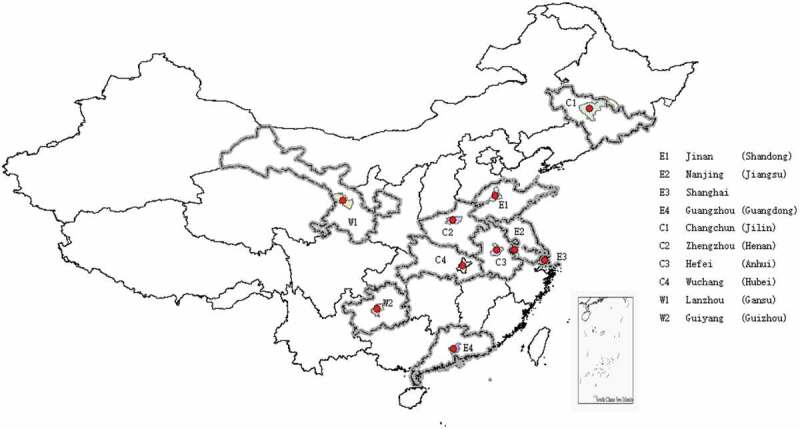
Sample distribution.

E, C and W means the eastern, central, and western regions of China, respectively. For E1, Jinan is the provincial capital city of Shandong province.

Much research in developed countries (U.S. and European countries) has investigated whether mandatory labeling affects consumer aversion to GM foods,^3,11-14^ consumers’ preference for GM food mandatory labeling,^15,16^ and limited usefulness of simple GM food labels.^10,13,17^ A consensus has been reached among researchers that consumers have a strong preference for GM food mandatory labeling.^15,16,18,19^ However, there are few similar findings in developing countries. China, as a developing country, enforced mandatory labeling for GM foods in 2004. Since then, most soybean oil for the Chinese consumers has been made from imported GM soybeans,^20^ and the Chinese soybean market has shifted from a Cournot-Nash equilibrium market to a Stackelberg equilibrium market.^21^ For instance, China imported about 90 million tons of soybeans in 2017, which were only used for processing materials. Most of the soybeans were produced in the U.S., Argentina, and Brazil,^22^ where GM soybeans are widely planted.^23^ Meanwhile, simple GM labels are adopted in China. Taking the directly processed GM agricultural products as an example, the label of GM soybean oil only shows “the raw material is GM soybeans.” Scholars indicate that the simple GM food labels are of limited usefulness.^10,13,17^ Nevertheless, it is even hard to find a GM label for the indirectly processed GM agricultural products with genetically modified organisms (GMOs) or the products containing agricultural GMOs as the raw materials.

Additionally, the public participation in the design of GM food labeling is very limited in China. Ascertaining the level of public support affects the implementation and success of mandatory labeling.^24^ However, there is a lack of systematic research on the preference of enhanced mandatory labeling or the public participation in the design of GM food labeling. To the best of the authors’ knowledge, no evidence on consumers’ preference for GM food labeling has been reported in China. Focusing on the mandatory labeling of GM foods, this paper aims to analyze Chinese urban consumers’ perceptions of traceability of GM soybean oil. The empirical evidence will provide scientific insights by estimating the effects of perceived attributes on the consumer preference for the mandatory labeling of GM foods.

## 2. Literature review

### 2.1. Consumers’ attitude toward GM mandatory labeling

Consumers’ attitudes toward GM mandatory labeling differ in different counties. Marchant and Cardineau^25^ outlined the debates among U.S. consumers on the labeling of GM foods. Public opinion polls have shown that more than 90% of U.S. consumers want GM foods to be labeled. Nep and O’Doherty^19^ found that consumers in British Columbia of Canada called for mandatory labeling of GM salmon (if approved for consumption), and their minimum requirement for labeling was to inform consumers if they were eating GM foods. Their results showed consumers’ demand for transparency in the production and governance of GM foods. Huang, et al.^26^ surveyed 400 consumers in Wuhan, China, and showed that 84.3% of the consumers agreed with the labeling of GM foods and they preferred the mandatory labeling. From a survey of 260 occupational groups across 11 provinces of China, Deng, et al.^27^ found more than 90% of the respondents believed that GM foods needed mandatory labeling. Further research in India also showed about 58% of Indian college students supported mandatory labeling of GM foods.^18^

Additionally, Teisl, Garner, Roe and Vayda^10^ indicated that a simple GM food label was of limited usefulness to American consumers. Examples including “whether to obtain GM ingredients” only allow consumers to differentiate the GM food products from their non-GM counterparts,^10^ while U.S. consumers were desired to see specific benefit and risk-related information on the GM food labels. On the contrary, excessive information presented on a GM food label may have a negative effect on the individuals who have less knowledge about genetic engineering and GM foods.^13^ Roe and Teisl^17^ conducted an experiment in which U.S. consumers were shown some sample labels with diverse claims regarding the presence of GM ingredients and the consumers then rated the labels with respect to the credibility and adequacy of the information content. They found that simple GM labels showing a product with GM ingredients were generally viewed more credible than simple non-GM labels showing a product not containing any GM content. However, most consumers believed the simple non-GM labels provided an adequate amount of information, rather than the simple GM labels. They also found the adequacy of the simple GM labels could be improved by mentioning the purpose of GM usage.

### 2.2. Determinants of consumers’ attitude toward GM mandatory labeling

Consumers’ attitudes toward GM mandatory labeling are influenced by multiple factors. Rimal, Moon and Balasubramanian^14^ used data covering 2568 samples from an online survey and showed that more than 75% of UK consumers were concerned about the labeling of food products with GM ingredients. They found the overall attitude toward GM labeling was shaped by five perceived risks associated with GM foods including health risk, environmental risk, moral consideration, concern of multinational corporations as the primary beneficiaries of biotechnology, and growing control of multinational corporations over farming systems. The only significant benefit from biotechnology, however, was found to be the improved nutritional content. Kajale and Becker^18^ conducted a survey among 298 Indian students and concluded that students who knew better about GM technology than the public were more likely to support the mandatory labeling of GM foods. Students’ dissatisfaction with current labeling policy and their demand for information about food production had a positive effect on their support for the mandatory labeling. Students who used food labels regularly were also more likely to support mandatory labeling.

Further, Zhao, et al.^28^ collected data from 1730 Chinese respondents including consumers, farmers, media and agricultural officials in relevant agricultural department. They examined the respondents’ attitude toward five different GM food labeling methods including no GM label, labels of meat fed by GM feeds, labels of cooking oil containing GM oil, labels of GM condiments, and labels of non-GM ingredients. The results showed that different groups of people had different attitudes toward GM food labeling methods, and those who were more familiar with GMOs or who trusted the government were more positive for GM labels. This result was similar to that of Kikulwe *et al*. (2013) that consumers who trusted government agencies better trusted the information from GM food labeling.

## 3. Theoretical framework and model

### 3.1. Traceability of GM soybean oil

Currently, mandatory labeling is enforced by many countries, such as the EU, China and the U.S. The provisions relating to the thresholds, exemptions, implementations and traceability are clearly different in different counties. The EU *traceability and labeling regulation 1830/2003296* and *Regulation (EC) on Novel Foods and Novel Food Ingredients* seeks to address the concerns about the lack of information to enable the labeling of GM foods. This policy sets out the requirements for a document audit trail to account for and identify approved GM products throughout the marketing chain. This regulation formulates the traceability requirements for food and feed produced from GM organisms should be established to facilitate accurate labeling of such products and enable the post-market monitoring on human health and environment.^29^

Chinese government also regulates the selection of enhanced mandatory labeling conveying some information about safety attributes.^[a]^^[a]^.*Regulations on Administration of Agricultural Genetically Modified Organisms Safety* and *Administrative Measures for Agricultural GMOs Labeling* issued by the Ministry of Agriculture and Rural Affairs of P. R. China. Based on the GM food sales and labeling requirements in China, the traceability is selected as one type of enhanced mandatory labeling. Following relevant literature^13,17,29-33^ and the representative GMO safety management policies, the traceability system has been established to document the entire process of GM soybean oil production in China. The system facilitates easy separation of the GM soybean oil from their non-GM counterparts “from farm to fork,” and well serves the purpose of marketing and health protection.

### 3.2. Consumer perception of traceability of GM soybean oil

It is assumed that a consumer is a rational economic man who allocates resources efficiently. Facing multiple options, the individual will choose the one that maximizes his or her expected utility. A consumer does not show a strong perception until the mandatory labeling can help maximize the utility. This study adopts the popular conceptual framework of attitude formation called Fishbein multi-attribute model, which has been used to analyze students’ opinions about the mandatory labeling policy of GM foods in India.^18^ According to the Fishbein model, the model in this research includes the perceived attribute of nutrition and potential health risk,^14,34,35^ perceived adequacy of simple GM labels^17^, and multiple control variables such as attention to food labels, and credibility of agency overseeing GMO safety.^28,36^ Socio-demographic factors^18^ are also included as explanatory variables to determine consumer perception on the traceability of GM soybean oil. The structure of the Fishbein multi-attribute model can be written as:

(1) $$Attitude_{i}=\sum_{j=1}^{n}\beta_{j}Attribute_{ji}+f\left(C,\lambda \right)+f\left(X,\gamma \right)+\mu_{i}$$

where *β_j_* are unknown regression coefficients, λ and γ are matrix of coefficients, *μ_i_* is a random disturbance term. *Attitude_i_* is the *i-th* consumer perception of the traceability of GM soybean oil. *Attitude_ij_* represents the perceived attributes including perceived attribute of nutrition and potential health risk, and perceived adequacy of simple GM label. *C* is a vector of control variables (i.e., attention to food labels, credibility of agency overseeing GMO safety). *X* is a vector of socio-demographic variables (i.e., gender, age, education attainment, child, occupation, and monthly per household disposable income).

### 3.3. Empirical model

Since the dependent variable (y) is binary (i.e., consumer perception of traceability = 1 if “need,” otherwise = 0), the logit model is suitable for this analysis. The general observation-level likelihood function is given by:

(2) $$\mathrm{Ln}L\left(\beta \left|y,x\right.\right)=\sum_{i=1}^{n}y_{i}\ln\left[\Lambda \left(x_{i}^{\prime }\beta \right)\right]+\sum_{i=1}^{n}\left(1-y_{i}\right)\ln\left[1-\Lambda \left(x_{i}^{\prime }\beta \right)\right]$$

where *β* is matrix of unknown regression coefficients, X is matrix of independent. The logit model with robust standard errors is conducted using the MLE estimation. All statistical and econometric analyses are conducted using STATA Version 16.

## 4. Data

### 4.1. Survey administration

Adhering to the stratified random sampling approach, this study used a self-administered questionnaire to collect data in the eastern, central, and western regions of China. Since the consumers’ perception may be different among different regions, we administered the survey in 10 provincial capital cities across the Chinese mainland. The eastern cities include Jinan, Nanjing, Shanghai and Guangzhou; the central cities include Changchun, Zhengzhou, Hefei and Wuchang; the western cities include Lanzhou and Guiyang. The survey was carried out in large-scale shopping malls and supermarkets. Consumers in those places are representative because they often decide which foods to buy for their family. Samples can also be collected among different social classes to avoid sample selection bias.^37,38^ We randomly selected consumers entering or exiting each market and followed sampling criteria including: the participants were urban residents and at least 18 years old; they had food purchase experiences; soybean oil was the main edible cooking oil in their families and was purchased rather than from squeezing on their own. Due to budget constraints, we cannot conduct the survey in all the cities of the selected provinces nor cover consumers in rural areas.

The survey was carried out by undergraduate students from Nanjing Agricultural University in February, 2017. The surveyors were trained before conducting the survey. Following procedures were followed: 1) The surveyor training focused on their understanding and interpretation of the survey questions. 2) A mock interview was conducted between two surveyors and feedback was used to improve the questionnaire. 3) A pilot survey was conducted, followed by a group discussion, which further helped improved the formal survey administration. Finally, the surveyors approached potential respondents and invited them to participate in the survey. Each respondent was offered a 10 RMB gift after they completed the interview.

To minimize the bias of acquiescence response, the questions were designed using Likert’ five-scale method. For instance, the possible options for the variable *consumer perception of traceability* were “completely do not need,” “do not need,” “neutral,” “need,” and “completely need” with a code from 1 to 5. Since the variable is categorical with unequal distances between any two categories, the variable could not be analyzed using the ordered logit model. We recoded a binary variable with “need=1” for “need” and “completely need,” and “otherwise=0” for “completely do not need,” “do not need,” and “neutral.” Additionally, the binary variable also facilitates easy understanding of the economic meaning of the variable. This method has been widely employed in agricultural economics, such as Yu et al. (2020).^7^ To minimize the bias of desirability bias, before filling out the questionnaire, the respondents were promised with the anonymity of the investigation and they were asked to complete the survey solely based on their own experiences. Moreover, the self-administered survey for data collection helps minimize the leading question bias and wording bias. This method also prevents the surveyors from summarizing what the respondents say or further taking what they say.

### 4.2. Data

The survey included questions about consumers’ perception of traceability, other influential factors, and socio-demographic inquiries. The respondents were provided with some detailed information on GM soybean imports and its connection to the public interest. The respondents were asked to answer questions about their perceived attributes, their attention to food labels, and their trust on the agency overseeing GMO safety. Then the respondents were asked about their perception of the traceability of GM soybean oil.

The construction of variables is built on the conceptual model of factors affecting consumer perception of traceability discussed above. Table 1 shows the variables and relevant survey questions. Each construct (i.e., perceived attribute of nutrition, perceived attribute of potential health risk, and perceived adequacy of simple GM labels) is calibrated with the response to each single question.

**Table 1.** Definition and descriptive statistics of variables.

Variables	Survey questions	Mean	Standard deviation
Consumer perception of traceability	Do you need the traceability of GM soybean oil? (Need = 1; otherwise = 0)	0.565	0.496
Perceived attribute of nutrition	Does GM technology improve the nutrition or quality of crops? (Agree = 1; otherwise = 0)	0.659	0.474
Perceived attribute of potential health risk	Do potential risks of GM foods pose to human because of eating this food in the long-term? (Agree = 1; otherwise = 0)	0.629	0.483
Perceived adequacy of simple GM labels	Whether the simple mandatory label, such as “the raw material is GM soybeans” on the label of GM soybean oil, provides adequate information to facilitate consumer choice?		
Inadequate	(Inadequate = 1; otherwise = 0)	0.236	0.425
Unclear	(Unclear = 1; otherwise = 0)	0.557	0.497
Adequate (base)	(Adequate = 1; otherwise = 0)	0.206	0.405
Attention to food labels	Do you look at the contents of the labels when purchasing foods? (Often look = 1; otherwise = 0)	0.376	0.484
Credibility of agency overseeing GMO safety	Do you trust in the agency overseeing GMO safety? (Distrust = 1; otherwise = 0)	0.279	0.448
Gender	Female = 1; male = 0.	0.499	0.500
Age	Middle-aged and elderly people (≥ 45) = 1; young people (18–44) = 0.	0.254	0.435
Education	Professional school, college degree or above = 1; senior high school or below = 0.	0.632	0.483
Child	Whether you have minors (≤ 15) at home? (Yes = 1; no = 0)	0.435	0.496
Occupation	Whether your work is related to biotechnology? (Yes = 1; no = 0)	0.079	0.271
Income	Monthly household disposable income.		
<2,000	2,000 RMB ^a^ or below = 1; otherwise = 0.	0.102	0.303
2,001–4,000	2,001–4,000 RMB = 1; otherwise = 0.	0.179	0.384
4,001–6,000	4,001–6,000 RMB = 1; otherwise = 0.	0.318	0.466
6,001–8,000	6,001–8,000 RMB = 1; otherwise = 0.	0.169	0.375
8,001–10,000	8,001–10,000 RMB = 1; otherwise = 0.	0.127	0.333
10,001–12,000	10,001–12,000 RMB = 1; otherwise = 0.	0.059	0.237
>12,000 (base)	12,000 RMB or above = 1; otherwise = 0.	0.045	0.207

^a^6.9 RMB = 1 USD.

Totally 850 respondents were surveyed. After accounting for missing observations, a total of 804 samples were included in this analysis (i.e., a valid return rate of 94.58%). Samples in the eastern, central and western regions accounted for 45, 35, and 20%, respectively.

## 5. Results and discussion

### 5.1. Descriptive statistics

Table 1 presents a statistic summary of the variables. The samples include less male and are better educated with 63.18% attending a professional school or holding a college or higher degree. The samples include more young people under 45 years old. About 8% of the respondents have a job related to biotechnology, and 56.47% of the families have minors (age ≤ 15). The average monthly household disposable income roughly follows a normal distribution, with categories 4,001–6,000 RMB, 6,001–8,000 RMB, and 8,001–10,000 RMB accounting for about 32, 17, and 13%, respectively.

The findings also show that 56.5% of the respondents required a need of traceability, 65.9% perceived the nutrition benefit, and 62.9% perceived the health risk. About 23.6% of consumers agreed that the simple GM soybean oil cannot provide adequate information for consumers, while 55.7% showed unclear. A majority (62.4%) of consumers often checked the contents of the label when buying food, which indicates that a substantial level of the Chinese consumers use the information on food labels. Most consumers (72.1%) trusted in the agency overseeing GMO safety.

A chi-square test is used to see if there is a relationship between two categorical variables. The results of Pearson chi-square test (see Table 2) suggest that consumer perception of traceability are associated with most variables including *perceived attribute of nutrition, perceived attribute of potential health risk, inadequate and unclear adequacy of simple GM labels, attention to food labels, and credibility of agency overseeing GMO safety*.

**Table 2.** Pearson chi-square test of independent variables.

Variable		Consumer perception of traceability		Pearson chi-square test
		Need = 1	Otherwise = 0	
Perceived attribute of nutrition	Agree = 1	322	208	11.628***
	Otherwise = 0	132	142	
Perceived attribute of potential health risk	Agree = 1	304	202	7.243**
	Otherwise = 0	150	148	
Perceived adequacy of simple GM label				
Inadequate	Inadequate = 1	128	62	12.027***
	Otherwise = 0	326	288	
Unclear	Unclear = 1	226	222	14.923***
	Otherwise = 0	228	128	
Adequate(base)	Adequate = 1	100	66	1.211
	Otherwise = 0	354	284	
Attention to foods label	Often look = 1	198	104	16.277***
	Otherwise = 0	256	246	
Credibility of agency overseeing GMO safety	Distrust = 1	144	80	7.721***
	Otherwise = 0	310	270	
Gender	Female = 1	231	170	0.422
	Male = 0	223	180	
Age	Middle-aged and elderly people (≥ 45) = 1	122	82	1.238
	Young people (18–44) = 0	332	268	
Education	Professional school, college degree or above = 1	294	214	1.110
	Senior high school or below = 0	160	136	
Child	Whether you have minors (≤ 15) at home?			
	Yes = 1	246	208	2.211
	No = 0	208	142	
Occupation	Whether your work is related to biotechnology?			
	Yes = 1	30	34	2.603
	No = 0	424	316	
<2,000	2,000 RMB ^a^ or below = 1	50	32	0.755
	Otherwise = 0	404	318	
2,001–4,000	2,001–4,000 RMB = 1	78	66	0.378
	Otherwise = 0	376	284	
4,001–6,000	4,001–6,000 RMB = 1	156	100	3.053*
	Otherwise = 0	298	250	
6,001–8,000	6,001–8,000 RMB = 1	66	70	4.196**
	Otherwise = 0	388	280	
8,001–10,000	8,001–10,000 RMB = 1	58	44	0.007
	Otherwise = 0	396	306	
10,001–12,000	10,001–12,000 RMB = 1	32	16	2.160
	Otherwise = 0	422	334	
>12,000 (base)	12,000 RMB or above = 1	14	22	4.738**
	Otherwise = 0	440	328	

### 5.2. Estimation results and discussion

The estimation results from the logit model are presented in Table 3. The condition number is 24.26 which is less than 30 and indicates no concern of multicollinearity. The Wald test (*p* <.001) suggests the logit model with robust standard errors provides valid estimation results.

**Table 3.** Estimation results of consumer perception of traceability of GM soybean oil using a logit model.

Variable	Coefficient	Standard error	Marginal effect	Standard error
Perceived attribute of nutrition	0.377***	0.140	0.080***	0.029

Perceived attribute of potential health risk	0.308**	0.137	0.066**	0.029
Inadequate	0.544**	0.219	0.116**	0.046
Unclear	−0.258	0.172	−0.055	0.037
Attention to food labels	0.428***	0.144	0.091***	0.030
Credibility of agency overseeing GMO safety	0.353**	0.157	0.075**	0.033
Gender	0.213	0.130	0.045	0.027
Age	0.523***	0.156	0.112***	0.033
Education	0.298**	0.142	0.064**	0.030
Child	0.409***	0.132	0.087***	0.028
Occupation	−0.425*	0.247	−0.091*	0.052
Income				
<2,000	1.148***	0.364	0.245***	0.077
2,001–4,000	0.790**	0.341	0.168**	0.072
4,001–6,000	1.158***	0.328	0.247***	0.069
6,001–8,000	0.597	0.333	0.127	0.071
8,001–10,000	0.683**	0.343	0.146**	0.073
10,001–12,000	1.442***	0.404	0.307***	0.085
City fixed effect	Yes			
Constant	−2.193***	0.452		
Fit statistics				
Number of observations	804			
Log pseudo likelihood	−741			
Wald	153***			
Pseudo R^2^	0.103			
Correctly classified	67.16%			

Robust standard errors are reported. *** *p* < 0.01, ** *p* < 0.05, * *p* < 0.1. Guiyang is the base category.

The variables *perceived attribute of nutrition* and *perceived attribute of potential health risk* show statistically significant and positive effects on consumer perception of traceability. The results indicate a positive relationship between consumer demand for traceability of GM soybean oil and their perceived attribute of nutrition and potential health risk. The more consumers perceive the risk and benefit of transgenesis and GM foods, such as the functions and potential health risk, the stronger their perceptions of traceability. The marginal effects indicate that consumers who perceive nutrition benefit are 8% more likely to support traceability, while consumers who perceive potential health risk are 6.6% more likely to support traceability. This result is similar with the findings of Rimal, Moon and Balasubramanian,^14^ Babasaheb Kajale and C. Becker^18^ and Zhao, Hu and Deng^28^ that consumers with better knowledge about transgenesis are more likely to support mandatory labeling of GM foods. Consumers’ perceptions about attributes related to GM foods are promoted by the new media that have been largely focused on reporting the controversy of GM foods. The perceptions are further increased by the scientific communication regarding the risks and benefits of transgenesis.

The perceived inadequacy of simple GM labels shows a positive effect, which indicates the inadequacy of simple mandatory labeling increases their demand for traceability. The marginal effect suggests that consumers who view the amount of information on simple GM labels as inadequacy are 11.6% more likely to support traceability of GM soybean oil. The result is similar to the finding of Kajale and Becker^18^ that students’ dissatisfaction with current labeling policy had a positive effect on their support for mandatory labeling. Rimal, Moon and Balasubramanian^14^ concluded that the amount of information on simple GM label was inadequacy. Because GM foods are subject to uncertainties such as potential health and ecological risks, simple GM food labels do not show much information and only helps consumers to differentiate GM foods from non-GM foods.^10^

Additionally, the consumers who pay more attention to food labels show a stronger demand for traceability of GM soybean oil. This result is consistent with the finding of Kajale and Becker^18^ that students who used food labels regularly were more likely to support mandatory labeling. Kornelis, De, Frewer and Dagevos^37^ showed that even for those “low users” who seemed to have a low interest in food-safety information, a low perception of information quality, and a low perceived health control, they were still very likely to use the safety information provided on the food labels. Zhang et al. showed that Chinese consumers’ opposition to GM technology was mainly due to their concerns about food safety.^39^ In fact, some food safety events in China, such as “big-headed children caused by milk power consumption” in 2003, “Sudan red eggs” in 2006, “butter oil” in 2010, just name a few, have shocked the public. Therefore, the Chinese consumers would like to pay more attention to food labels.

The variable *credibility of agency overseeing GMO safety* shows a positive impact. It indicates that those who do not trust government agencies overseeing GMO safety show a stronger demand for traceability. Numerous studies have indicated that confidence in the government management of biotechnology plays a critical role in the attitudes toward GM foods and commercialization of GM crops.^7^ For example, Zhao et al.^40^ indicated that confidence in the government management of GM food labeling significantly influenced Chinese consumers’ attitudes toward labeled GM products. However, our results show more than four fifths of consumers have no confidence in the government management of biotechnology. Deng et al.^41^ indicated that this distrust might be because consumers do not know the great efforts that the government has made in the management of biotechnology. For instance, China has developed a comprehensive biotechnology policy and regulatory system which guides the biotechnology development.

Furthermore, the effect of having children is positive, which indicates respondents with minors at home have a better perception of traceability of GM soybean oil. More individual characteristics also show significant marginal effects. For example, the consumers who are well educated and older show a greater demand for traceability, while those whose jobs are related to biotechnology show a lower demand. Household disposable income is also a key factor, and consumers from the highest income group show a lower demand. This may be because the wealthier consumers are more concerned about their health with GM food consumption. Therefore, they are more likely to buy the non-GM soybean oil instead of the GM counterpart.

### 5.3. Heterogeneity check

Based on the above estimation results, adequacy of simple mandatory labeling is a key factor to consumers’ preference for traceability. This section discusses the robustness of marginal effects by groups according to the perceived information adequacy on the simple mandatory label. The robustness results are shown in Table 4.

**Table 4.** Marginal effects from the robustness check using different categories of the information adequacy of the simple labels.

Variable	Inadequate = 1	Unclear = 1	Adequate = 1
Perceived attribute of nutrition	−0.094	0.070	−0.144
	(0.073)	(0.037)	(0.092)
Perceived attribute of potential health risk	0.123**	0.139***	−0.114
	(0.058)	(0.036)	(0.060)
Attention to food labels	0.008	0.071	0.349***
	(0.059)	(0.041)	(0.057)
Credibility of agency overseeing GMO safety	0.065	0.113**	0.096
	(0.062)	(0.049)	(0.070)
Control variables	Yes	Yes	Yes
Socio-demographic variables	Yes	Yes	Yes
Fit statistics			
Observations	190	448	166
Log pseudo likelihood	−145.968	−366.678	−111.973
Wald	69.44***	153.31***	74.41***
Pseudo R^2^	0.167	0.213	0.331
Correctly classified	73.63%	73.21%	79.52%

Robust standard errors in the parentheses. *** *p* < 0.01, ** *p* < 0.05.

For the consumers who view the simple mandatory label as inadequate, the variable *perceived attribute of potential health risk* shows a positive effect. The marginal effect indicates that consumers who perceive attribute of potential health risk are 12.3% more likely to support traceability. For consumers with unclear adequacy, the variables *perceived attribute of potential health risk* and *credibility of agencies overseeing GMO safety* show positive impacts. Further, the results show a positive relationship between consumer demand for traceability of GM soybean oil and their perceived attribute of potential health risk and their distrust in agency overseeing GMO safety. Additionally, for the consumers with an adequate attitude, the variable *attention to food labels* shows a positive influence. This indicates even though those consumers may be satisfied with the simple mandatory labels, these who often look at the contents of the labels when purchasing food are 34.9% more likely to support traceability of GM soybean oil. These results are consistent with the findings from the above baseline model.

## 6. Conclusions and policy implications

This study assessed Chinese urban consumers’ perception of traceability of GM soybean oil and examined the effects of perceived attributes. We obtain unique and interesting results that can provide insights for policymakers, stakeholders, and practitioners. Firstly, the findings show that more than half of the consumers show preference of traceability of GM soybean oil. The results of the logit model suggest the consumer perception of traceability are affected by perceived attributes of nutrition and potential health risk, and perceived adequacy of the simple GM label. The urban residents have a stronger perception of traceability of GM soybean oil if they perceive attributes of nutrition and potential health risk of GM foods. The inadequacy of simple mandatory labels also shows a positive impact. The results signify that Chinese consumers are aware of the significance of traceability of GM foods.

Efforts from government to enhance mandatory labeling and strategize relevant programs may benefit the urban residents. Agencies primarily undertake the costs of improving the management of GM food mandatory labeling, and public-involved policies are critical to gain more consumer support. Especially, consumers will perceive more attributes about GM foods in the future because of the great influence of new media and scientific communication regarding the risks and benefits of the transgenesis. Therefore, policies should be designed from the perspectives of both policymakers and consumers. Relevant policies should not only fully respect consumers’ right-to-know, but also facilitate a rational purchase decision on edible oil. In addition, some actions should be taken to promote the credibility of the agencies. For instance, GM food mandatory labels should be examined regularly; any illegal non-GM food labels should be prohibited; and false or exaggerated labels should be corrected. Strict penalty mechanisms should be enforced on the food companies that violate the policies and public media can widely inform consumers if any food safety problems are found. Regular updates should be made regarding the types of GM foods in the market, the catalog of imported GM foods, and the GMO safety certification for commercial planting.

There are opportunities for some future research. This study only focuses on soybean oil made from GM soybeans. Although there is, as of yet, no mandatory labeling requirements for other GM foods expect soybean oil and rapeseed oil in China, this approach can be applied to other GM foods later. Additionally, better understanding the traceability of GM soybean oil requires scientific efforts on more relevant topics, such as consumers’ willingness-to-pay for traceability of GM foods. Future research can also examine consumers’ attitude toward GM food labeling and other concerns about food safety.
